# An enhanced level of VCAM in transplant preservation fluid is an independent predictor of early kidney allograft dysfunction

**DOI:** 10.3389/fimmu.2022.966951

**Published:** 2022-08-11

**Authors:** Michael Baboudjian, Bastien Gondran-Tellier, Romain Boissier, Patricia Ancel, Juline Marjollet, Luc Lyonnet, Pauline François, Florence Sabatier, Eric Lechevallier, Anne Dutour, Pascale Paul

**Affiliations:** ^1^ Department of Urology and Transplantation, La Conception Hospital, Assistance Publique-Hôpitaux Marseille, Marseille, France; ^2^ Department of Urology, Assistance Publique-Hôpitaux de Marseille, Hopital Nord, Aix-Marseille University, Marseille, France; ^3^ Institut national de la santé et de la recherche médicale (INSERM) 1263, Aix Marseille University, French national research institute for agriculture, food and the environment (INRAE), Centre de recherche en CardioVasculaire et Nutrition (C2VN), Marseille, France; ^4^ Department of Hematology, Hopital de la Conception, Assistance Publique-Hôpitaux Marseille, Marseille, France; ^5^ Cell Therapy Laboratory, Centre d'Investigation Clinique (CIC)-149, La Conception Hospital, Assistance Publique-Hôpitaux Marseille, Marseille, France; ^6^ Endocrinology, Metabolic Diseases and Nutrition Department, Assistance Publique Hôpitaux de Marseille, Marseille, France; ^7^ Institut national de la santé et de la recherche médicale (INSERM) unité mixte de recherche (UMR)_1090, Aix Marseille University, TAGC Theories and Approaches of Genomic Complexity, Parc Scientifique de Luminy Case 928, Marseille, France

**Keywords:** extended criteria donor, delayed graft function, machine perfusion, VCAM = vascular cell adhesion molecule, kidney transplantation

## Abstract

**Background:**

We aimed to evaluate whether donor-related inflammatory markers found in kidney transplant preservation fluid can associate with early development of kidney allograft dysfunction.

**Methods:**

Our prospective study enrolled 74 consecutive donated organs who underwent kidney transplantation in our center between September 2020 and June 2021. Kidneys from 27 standard criteria donors were allocated to static cold storage and kidneys from 47 extended criteria donors to hypothermic machine perfusion. ELISA assessment of inflammatory biomarkers (IL-6, IL6-R, ICAM, VCAM, TNFα, IFN-g, CXCL1 and Fractalkine) was analyzed in view of a primary endpoint defined as the occurrence of delayed graft function or slow graft function during the first week following transplantation.

**Results:**

Soluble VCAM levels measured in transplant conservation fluid were significantly associated with recipient serum creatinine on day 7. Multivariate stepwise logistic regression analysis identified VCAM as an independent non-invasive predictor of early graft dysfunction, both at 1 week (OR: 3.57, *p* = .04, 95% CI: 1.06-12.03) and 3 Months (OR: 4.039, *p* = .034, 95% CI: 1.11-14.73) after transplant surgery.

**Conclusions:**

This prospective pilot study suggests that pre-transplant evaluation of VCAM levels could constitute a valuable indicator of transplant health and identify the VCAM-CD49d pathway as a target to limit donor-related vascular injury of marginal transplants.

## Introduction

Kidney transplantation is the optimal treatment for most patients with end-stage kidney disease with benefits for both quality and quantity of life (1, 2). In spite of considerable progress in improving graft survival after kidney transplantation, the issues that interfere with successful outcome remain unresolved. One is the growing discrepancy between the availability of organ donation and the increasing need for kidney grafts associated with a rising incidence of end-stage renal disease in elderly patients (3). Increasing recipient demand combined with inadequate organ supply has led to the use of suboptimal marginal kidneys from expanded criteria donors (ECD) with cardiovascular risk factors (4, 5). However, ECD kidney transplants have been associated with worsened outcomes and higher rates of early graft dysfunction – defined as delayed (DGF) or slow graft function (SGF) – compared with standard criteria donors (SCD) (6). Prediction of early graft dysfunction is a major concern since it is associated with post-transplant renal complications, higher incidence of acute rejection, poorer long-term graft survival, and higher costs of kidney transplantation (7, 8).

Several attempts have been made to identify recipient biomarkers that can predict early graft dysfunction (7, 9, 10).However, recipient biomarkers are difficult to assess in the decision to allocate deceased-donor kidneys in current clinical practice. A definition of donor-related biomarkers in kidney transplant biopsies or in serum or urine fluids could be a valuable tool to assess the pre-transplant quality of kidneys and orient early management of risk after transplant surgery (11, 12).These biomarkers are limited by the accessibility of donor-derived fluid or procurement biopsies during the clinical transplantation procedure (10, 13, 14).

Static cold storage of donor organs remains the common method to preserve solid organs. The concept of hypothermic machine perfusion (HMP) of deceased-donor kidneys provides an attractive alternative to limit transplant vascular injury prior transplant (15–17). To date, only a few studies have investigated whether soluble markers placed in kidney preservation solutions could reflect transplant quality (10). Our working hypothesis was that the paracrine secretion of markers from the graft could represent a non-invasive and timely source of donor material with potential value to reflect the inflammatory features that impact transplant quality and function. This prospective pilot study was therefore designed to investigate whether quantification of pro-inflammatory cytokine and chemokine levels in the kidney transplant preservation solution during transplant cold storage of organs can anticipate early kidney transplant dysfunction.

## Methods

### Study design and participants

This prospective single-center cohort study was conducted between September 2020 and June 2021 in accordance with the principles of Good Clinical Practice and the Declaration of Helsinki. The study protocol was approved by the National Ethics Committee of the *Agence de la Biomédecine* (PFS18-013), the National Ministry of Research and adhered to the Jardé Law on human investigation. Eligible patients were aged 18 years or over, had a negative cross-match and included all deceased-donor kidney transplant recipients. Kidney transplants from living donors and transplant donation after circulatory death were excluded from the study. A total of 77 donors were initially enrolled. Three patients requiring early allograft nephrectomy owing to arterial (n = 2) and venous thrombosis (n = 1) were excluded from the final analysis. From these sources, donor and recipient risk factors commonly associated with outcomes were prospectively collected. Donor factors included patient age and gender, body mass index (BMI), blood group, hypertension, comorbidities, serum creatinine level (µmol/L), donor criteria SCD or ECD criteria, cold ischemia time, and type of organ preservation (static cold storage or HMP).

### Renal transplantation

After organ removal, kidneys from SCD were allocated to static cold storage and were submerged in Institut Georges Lopez-1 solution (Institut Georges Lopez-1 solution IGL-1, Lissieu, France) at 4°C, according to established Eurotransplant guidelines. The kidneys from ECD allocated to hypothermic machine perfusion were placed on the Organ Recovery Systems LifePort (LifePort—Organ Recovery System or Waves machines—Waters Medical Systems) devices using kidney preservation solution 1 (KPS-1). Renal resistances were monitored and flow adjusted, but none of the kidneys was discarded based only on intravascular resistance and flow measurements. All transplantations were performed through an open and extraperitoneal approach. The renal vein and renal artery were anastomosed end-to-side to the external iliac vessels. Extravesical ureteroneocystostomy was created according to the Lich-Gregoir technique. Double-J^®^ stent was routinely placed in the ureter to protect the anastomosis and was endoscopically removed after 14 days. All of the patients received the same induction therapy: rabbit anti-thymocyte globulin (Thymoglobuline^®^) was administered on day 0 (1.25 g/kg/day) for 8 days, prednisolone administration on day 0 (initially 1 mg/kg/day), with subsequent tapering to achieve a targeted mean maintenance dose of 0.25 mg/kg/day on day 30 after transplant. Two types of immunosuppressive regimen were given as maintenance treatment during the study period: Tacrolimus, mycophenolate mofetil (FK/MMF) and corticosteroid or cyclosporin, azathioprine (CSA/Aza) and corticosteroids.

### ELISA evaluation of inflammatory biomarkers in the transplant preservation solution

Immediately before transplantation, 50 mL of preservation solution was collected, stored at 4°C, centrifuged at 400g for 5 minutes in order to remove the cellular debris. Samples were aliquoted and stored at -80°C. Quantitative assay of interleukin 6 (IL-6), IL-6 receptor (IL-6R), tumor necrosis factor (TNFα), intercellular adhesion molecule (ICAM), vascular cell adhesion molecule (VCAM), C-X-C motif chemokine ligand 1 (CXCL1), interferon-γ (IFNγ), and chemokine C-X3-C motif ligand 1 (CX3CL1/Fractalkine) was performed on the thawed conservation fluids, according to manufacturer instructions using Quantikine^®^ ELISA kits (R&D systems, Minneapolis, USA).

### Analysis of angiogenic activity and inflammatory cells that reside in the stromal vascular fraction of the transplant perirenal adipose tissue (PRAT-SVF)

Angiogenic parameters previously evaluated on PRAT-SVF in a subgroup of 17 donors (9 ECD versus 8 standard criteria donors) (18), were used to analyze the potential correlation with soluble inflammatory markers evaluated in the perfusate of corresponding donors. Various parameters reflecting the relevant steps of SVF vasculogenic/angiogenic activity (19) were quantified: the number of clusters, indicative of the capacity of the plated cells to self-assemble; the number of clusters with tip cells, indicative of the ability of cells to undergo specialization into cells able to migrate away from the cluster and initiate sprouting; the number of clusters with stalk cells that represent the capacity of cells to proliferate and elongate neovessels; and the number of branching points that provide information on the capacity of cells to develop as complex vascular networks. Multiparameter flow cytometry analysis of the distribution of the CD45- and CD45+ cell subsets donor-derived in PRAT-SVF was previously obtained on the same 17 donors (18).

### Study endpoints

The primary endpoint was early graft dysfunction defined as the occurrence of delayed graft function (DGF, defined as the requirement for dialysis during the first week after transplantation) or slow graft function (SGF) defined by recipient serum creatinine > 250 µmol/L (3.0 mg/dL) on postoperative day 7.

Secondary endpoints included predictors of renal dysfunction 3 months following transplantation (DGF <45mL/min/1.73 m^2^) (measured by creatinine and by glomerular filtration rate as estimated by the chronic kidney disease epidemiology collaboration (CKD-EPI) equation).

### Statistical analysis

Statistical analyses were performed using R statistical software Version 4.0.2. (Foundation for Statistical Computing, Vienna, Austria) and GraphPad Prism v9.0.2 software (San Diego, CA, USA) for graphical representation of figures. Descriptive statistics were conducted on variables described in **Table 1**. Quantitative variables were reported as medians and interquartile ranges [IQR 25-75] and analyzed using a non-parametric Mann-Whitney test. Categorical variables were described by numbers and percentages and analyzed by Fisher’s exact test and chi-square test as appropriate. Spearman’s correlation test was used to assess the relationship between VCAM and other variables.

**Table 1:** Donor baseline characteristics.

	Overall Cohort	SCD	ECD	*p-value*
	(n = 74)	(n = 27)	(n = 47)	
**Age, *years***	58.5 (47-69)	47 (34-54)	67 (58-74)	<.001***
**Gender, *n (%)***				.015*
Male	44 (59.5)	21 (77.8)	23 (48.9)	
Female	30 (40.5)	6 (22.2)	24 (51.1)	
**BMI, n, (IQR)**	25 (23-29)	25 (22-29)	26 (24-30)	.12^t^
**Arterial hypertension, *n (%)***	29 (39.2)	2 (7.4)	27 (57.4)	<.001***
**Dyslipidemia, *n (%)***	3 (4)	0 (0)	3 (6.4)	.29, ns
**Diabetes mellitus, *n (%)***	5 (6.8)	4 (14.8)	1 (2.1)	.06 ^t^
**Coronary disease, *n (%)***	6 (8.1)	1 (3.7)	5 (10.6)	.40, ns
**History of stroke, *n (%)***	22 (29.7)	5 (18.5)	17 (36.2)	.12 ^t^
**Creatinemia, *µmol/L, (IQR)***	64 (48-82)	68 (49-82)	62 (49-80)	.60, ns
**Cold ischemic time, *min, (IQR)***	778 (570-957)	812 (585-938)	778 (566-1018)	.96, ns
**Transplant storage, n**				<.001***
Static	35	27	8	
HMP	39	0	39	

SCD, standard criteria donors; ECD, extended criteria donors, BMI, body mass index; HMP, hypothermic machine perfusion. Quantitative variables correspond to % when stated or median values and interquartile ranges (IQR: 25-75 interquartile ranges). Significant p values were noted using asterisk: *** p ≤.001. p values >.05 and <.2 are noted as ^t^ or ns, non significant (p>.2); Results are expressed as median and 25-75% interquartile ranges or proportion (%).

To assess the predictors of DGF/SGF in the overall cohort and in the ECD cohort, we performed a multivariate logistic regression analysis using a stepwise backward elimination step allowing evaluation of adjusted odds rations (ORs) and 95% confident intervals (CIs). All tests were two sided, and.05 was considered as significant.

## Results

### Study population

**Figure 1:**
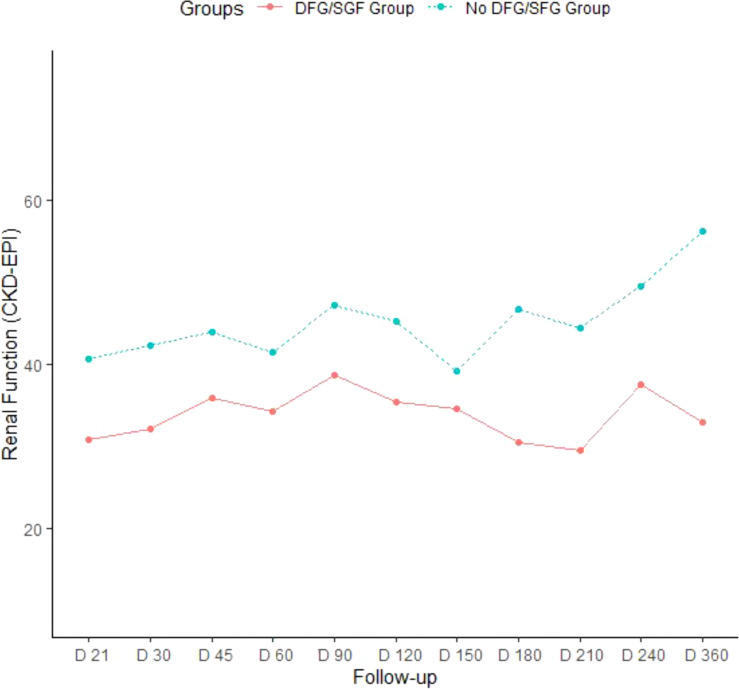
Evolution of renal function according to the occurrence of early ECD transplant dysfunction 7 days post-transplant surgery eGFR (mL/min/1.73 m^2^) was analyzed in kidney transplant recipients at different time points (D: days) and notably at two months and 3 months following transplant surgery within the group of the 47 ECD donors (Extended Criteria Donors), further stratified according to the occurrence or delayed or slow graft dysfunction during the first week following transplantation (DGF/SDG group, lower line) or no early graft dysfunction at day 7 (upper dot line).

A total of 74 donor fluids and recipients were included: 27 grafts from optimal donors (or standard criteria donors referred to as SCD) and 47 from marginal donors (extended criteria donors or ECD). Donor baseline characteristics are summarized in **Table 1**. SCD and ECD were compared in terms of demographic characteristics, comorbidities, and cold ischemia time. Static cold storage was used for all SCD grafts. In the ECD group, eight grafts were not preserved using HMP owing to technical issues related to renal artery cannulation. Median cold ischemia time was similar between both groups. Among the donor parameters analyzed, only male gender (18/25 patients, *p* = .117) and donor obesity (BMI>30, *p* = .094) tended to be associated with early graft dysfunction by day 7 post-transplant. When considering the ECD group only (n = 47), 36% (17/47) of the donors experienced delayed or slow graft function during the first week following transplantation. While donor age, gender or dyslipidemia did not significantly impact early ECD graft dysfunction, the occurrence of DGF/SGF by day 7 post-transplant tended to be associated with donor obesity (*p* = .064) and HTA (*p* = .170). Creatinine levels on day 7 showed a strong correlation with creatinine levels evaluated at one month, 2 months and 3 months post-transplant and were inversely correlated to the CKD-EPI of transplant recipients at 3 months post-transplant (**Supplemental Table 1**). Patients with early graft dysfunction (DGF/SGF) by day 7 had significantly lower estimated glomerular filtration rate (eGFR) on day 30 (*p* = .008), day 45 (*p* = .005), and day 180 (*p* = .003) post-transplant (**Figure 1**).

### Comparative analysis of soluble inflammatory biomarkers in the organ preservation fluid of optimal and marginal donors

**Figure 2:**
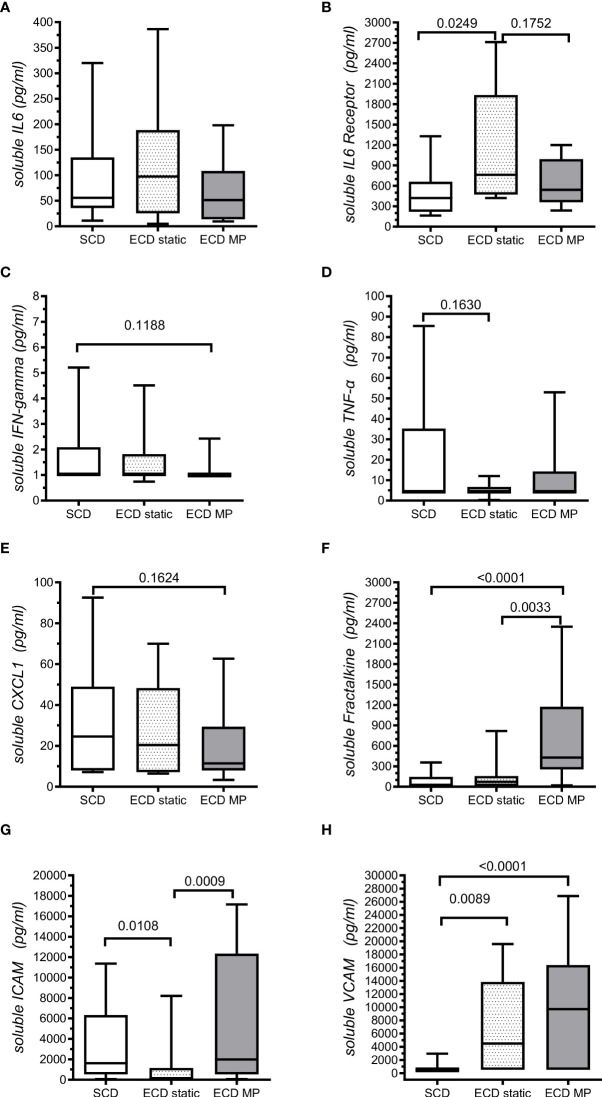
Quantitative analysis of soluble inflammatory biomarker levels in transplant preservation fluids. Levels of soluble iammatory markers were analyzed by ELISA in preservation fluids from SCD transplants (Standard criteria donors n = 27, all conserved in static conditions) and from ECD transplant (extended criteria donorsn n=47) further classified as a group of transplants conserved in static conditions (ECD static: n = 8) and a group of transplants conserved by machine Perfusion (ECD MP, n = 39). Different panels of soluble markers evaluated are represented : **(A)** Soluble IL6, **(B)** Soluble IL6 receptor, **(C)** Soluble IFN-gamma, **(D)** Soluble TNF-alpha, **(E)** Soluble CXCL1, **(F)** Soluble Fractalkine, **(G)** Soluble ICAM, **(H)** soluble VCAM. Significant p values (*p* <.05) or trends for significance were (*p* < .2) resulting from non- parametric Mann-Whitney test comparison of evaluated soluble biomarkers median levels are indicated.

**Figure 3:**
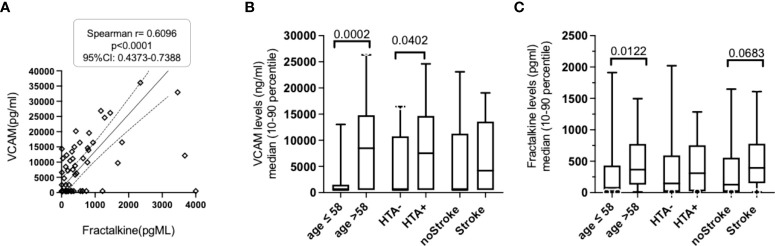
Analysis of fractalkine and VCAM levels association with ECD donor-related risk factors. **(A)**. VCAM levels were significantly correlated with the fractalkine levels evaluated in the perfusion fluid of ECD donors. **(B)**. Comparative analysis of VCAM levels in perfusion fluid of transplant stratified according to three donor-related risk factors (donor age > 58.5 years, HTA and donor stroke) revealed that VCAM levels were significantly higher in aging ECD donors (> to the median 58 years value observed in the cohort and in conservation fluids of transplant from donors with hypertension (HTA). Significant *p* values (*p* <.05) or trends for significance were (p < .2) resulting from non-parametric Mann-Whitney test comparison of evaluated soluble biomarkers median levels are indicated. Non-significant values >.2 are not reported. **(C)**. Comparative analysis of fractalkine levels in the perfusion fluid of transplants stratified according to three risk factors (age >58years, HTA and donor stroke) revealed enhanced levels in aging donors and a trend for enhanced fractalkine levels in preservation fluid in deceased donors with stroke.

The comparative analysis of inflammatory biomarkers evaluated in the preservation fluids from ECD and SCD donors is presented in **Supplemental Table 2**. The levels of IL-6, TNFα, ICAM, CXCL1 and IFNγ were comparable in SCD and ECD donors. In contrast, the level of VCAM and Fractalkine/CX3CL1 levels were observed to be significantly higher in the perfusion fluid of ECD donors analyzed in reference to SCD. A trend (*p* <.1) for enhanced soluble IL-6 receptor and lowered IFNγ levels was also observed in the ECD group but did not reach significance. When further discriminating ECD, donors conserved in static or perfused conditions (**Figures 2A–H**), IL6-R levels were found to be significantly higher in the preservation fluid of ECD donors conserved in static conditions (median value: 761 pg/mL) when compared with SCD donors (median value: 420 pg/mL) and tended to be higher than the values observed in ECD donors conserved in HMP (541ng/mL, **Figure 2B**). Median values of Fractalkine/CX3CL1 (**Figure 2F**) and ICAM (**Figure 2G**) were observed as enhanced in ECD patients under HMP (median Fractalkine 447pg/mL) when compared with ECD conserved in static conditions. In contrast, regardless of the transplant conservation procedure, the VCAM median levels observed in ECD donors conserved with HMP (n = 39, median 9720 pg/mL) or in static conditions (n = 8, median 4490pg/mL) were observed to be significantly higher when compared with SCD donors (n = 27, median 500pg/mL, **Figure 2H**). On analysis of the entire cohort, a positive correlation was observed between VCAM levels and other inflammatory biomarkers such as fractalkine, ICAM and IL-6R (**Table 2**). However, on analysis of ECD versus the SCD donor groups, we noted that a significant correlation between Fractalkine and VCAM inflammatory markers evaluated in the perfusate was only retained for donors with the ECD status conserved with machine perfusion (**Figure 3A**). When limiting analysis to the ECD group of donors, this level of fractalkine was also significantly correlated with that of IL6-R (r = 0.277, *p* = .0167), TNF-α (r = 0.375, *p* = .0095) and ICAM (r = 0.354, *p* = .0146).

**Table 2:** Correlation observed between sVCAM levels and other inflammatory biomarkers in the entire cohort.

Variable	*Spearman r*	*p value (two-tailed)*
Donor age	0.4158	.0002
Cold ischemia duration	0.2943	.0109
***Kidney allograft function***	*** ***	*** ***
Creatinine day 7	0.3966	.0005
Creatinine 1 month post-transplant	0.4041	.0004
CKD 1 month post-transplant	-0.4504	<.0001
CKD 3 months post-transplant	-0.4128	.0008
***Soluble inflammatory molecules in conservation fluid***	*** ***	*** ***
Fractalkine	0.6096	<.0001
CXCL1	-0.7037	.0033
ICAM	0.2972	.0101
IL-6 receptor	0.2465	.0342
Angiogenic activity of PRAT-SVF cells*	*** ***	*** ***
matrigel: number of branching points	-0.7102	.0021
% of clusters with stalk cells	-0.6912	.003
% of clusters with tip cells	-0.6782	.0037
Number of matrigel Clusters	-0.4961	.0448
Inflammatory infiltrate PRAT-SVF cells *		
CD3-CD56+ NK cell infiltrate	0.5275	.0316

PRAT, PeriRenal Adipose Tissue; SVF, stromal vascular fraction. p values correspond to significant correlation (p <.05). *Correlation of VCAM levels evaluated in preservation fluid with angiogenic and inflammatory parameters obtained from a previous study on a subgroup of 17 donors (9 ECD and 8 standard donors).

### Analysis of the association between soluble inflammatory molecules and donor characteristics

When analyzed in the entire cohort, donor age was correlated with VCAM levels (r=0.416, *p*=0002, **Table 2**). Enhanced fractalkine levels (**Figure 3B**) and VCAM levels (**Figure 3C**) were also associated with donor aging (> the 58 years median value observed in the entire cohort). Donor HTA (n = 29) was found to be associated with enhanced VCAM levels (median 7534 versus 500 pg/mL in the HTA-free group of donors, *p* = .040). In contrast, other donor characteristics such as gender, BMI or obesity (BMI>30), dyslipidemia, diabetes mellitus and coronary disease were not statistically associated with the values of the inflammatory molecules evaluated in the entire cohort. In addition, significant associations were found between increased cold ischemia duration and some of the evaluated biomarkers evaluated in the conservation fluid obtained from the entire cohort: VCAM (r = 0.294, *p* = .0109), ICAM (r = 0.261, *p* = .0244), IL6-R (r = 0.261, *p* = .025), and fractalkine (r = 0.237, *p* = .042) levels.

### Predictors of early graft dysfunction

We further analyzed whether evaluation of the levels of inflammatory molecules in the machine perfusion fluid of ECD transplants could be associated with the occurrence of early graft dysfunction during the first week following transplant surgery. Among the machine perfused ECD transplant (n = 39), IL6 and VCAM were the only soluble inflammatory markers that were significantly different in the perfusion fluid of ECD donors according to early dysfunction of the graft (**Table 3**).

**Table 3:** Inflammatory biomarker levels observed in the machine perfusion fluid of ECD transplants and delayed/slow graft function.

	ECDNo DGF (n = 26)	ECDDGF/SGF at day7 (n = 13)	*p*-value
**IL-6, *pg/mL***	20 (11-101)	87 (30-126)	.493
**IL-6R, *pg/mL***	532 (337-954)	562 (217-1262)	ns
**Ratio IL-6R/IL-6**	14 (5-55)	12 (3-18)	.1244^t^
**TNF*α*, *pg/mL***	4 (4-17)	4 (4-41)	ns
**ICAM, *pg/mL***	1960 (443-9650)	4451 (621-15965)	.1700^t^
**VCAM *pg/mL***	4187 (500-11369)	16506 (10584-29904)	<.0001
**CXCL1, *pg/mL***	12 (8-28)	11 (6-52)	ns
**IFN*γ*, *pg/mL***	1 (1-2)	1 (1-3)	ns
**Fractalkine, *pg/mL***	374 (173-836)	771 (416-2076)	.0630^t^

ECD: extended criteria donors. DGF/SGF: delayed graft or slow graft function by day 7 post-transplant. Analysis was restricted to the group of ECD donors whose transplants were conserved under machine perfusion (n = 39). Indicated values correspond to median and 25-75 Interquartile ranges. Median values observed in SCD and ECD donors were considered significantly different using non-parametric Mann-Whitney Tests when p values were <.05. p values <.2 were noted ^t^ as a trend for significance. ns: p value >.2

Quantitative analysis of serum creatinine on day 7 was significantly correlated with the level of VCAM (r = 0.397, *p* = .0005), recipient age (r = 0.313, *p* = .0067), recipient BMI (r = 0.301, *p* = .0091) and tended to be inversely correlated to the IL-6 Receptor/IL-6 ratio evaluated in conservation fluids (*p* = .1639). The VCAM levels evaluated in transplant conservation fluids were also correlated to creatinine levels evaluated in transplant recipients at 1 month post-transplant (**Table 2**).

Consistently, renal function evaluated 3 months after transplantation was also inversely correlated with VCAM levels (r = -0.411, *p* <.001, **Table 2**) and IL6 levels (*p* = .7407, *p* = .0034) in preservation fluid, recipient age (r = -0.606, *p* <.001), recipient BMI (r = -0.3248, p = .009), and donor age (r=-0.5837, *p* <.001).

### Enhanced VCAM levels in conservation fluid can be identified as independent predictor of early graft dysfunction

**Figure 4:**
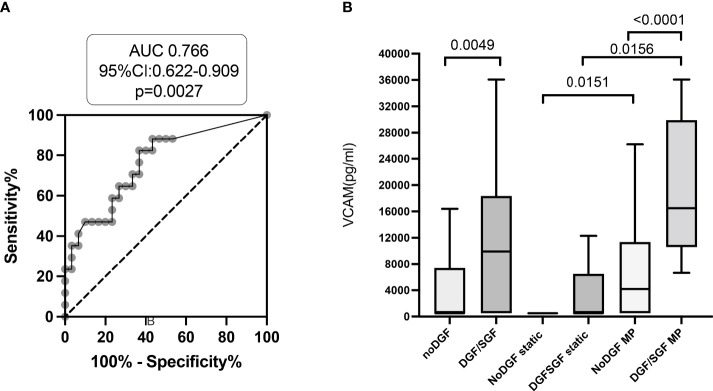
VCAM levels evaluated in transplant conservation fluid associate with early graft dysfunction. **(A)**. Receiver operating characteristic analysis of the value of VCAM levels predicting early graft dysfunction of ECD transplants at 7 days post-transplant surgery. Area under the curve (AUC) and 95% confidence intervals of prediction models are indicated. **(B)**. Enhanced VCAM values are observed in conservation fluid of machine-perfused transplants with delayed or slow graft dysfunction by day 7. *p* values were considered significant at <.05.

ROC curve analyzed VCAM levels accuracy to predict early graft dysfunction at 1 week post-transplant with an area under the curve of 0.684 (95%CI: 0.547-0.821, *p* = .0101) for the entire cohort and 0.766 (95% CI, 0.622-0.909, *p* = .027) when considering the ECD group (**Figure 4A**). Maximal sensitivity and specificity of VCAM corresponded to a value of 8008 pg/mL and were respectively 82% and 63%. Univariate logistic regression analysis revealed that VCAM levels were significantly enhanced in machine-perfused ECD donors with early graft dysfunction (**Figure 4B**). Multivariate stepwise logistic regression analysis including donor characteristics that tend to associate with delayed or slow graft function in univariate analysis revealed that among inflammatory markers evaluated in conservation fluid, a VCAM level above the 550 pg/ml threshold was the only soluble molecule that retained value to predict early kidney transplant dysfunction by day 7 post-transplant, both in the entire cohort or when restricting analysis to the ECD group of transplants (**Table 4**).

Table 4Univariate and multivariate analysis of donor variables associated with early allograft dysfunction by day 7 post-transplant and 3 months post-transplant.

**Table:** 

*Early kidney graft dysfunction (DGF or SGF) n = 74*	*Univariate analysis*		*Multivariate logistic regression*				
	*OR*	*p*	*OR*	*p*	*95% CI*		
VCAM > 550*pg/ml*	2.8	.043	3.57	.040	1.06	–	12.03
Donor gender male	2.27	.121	3.17	.049	1.00	–	10.03
Donor BMI >30	2.5	.100	3.09	.070	0.91	–	10.43
Donor age >58 years	1.44	.462	1.09	.880	0.32	–	3.73

**Table:** 

*Early kidney graft dysfunction (DGF or SGF) ECD* n = 47		*Univariate analysis*		*Multivariate logistic regression*				
		*OR*	*p*	*OR*	*p*	*95% CI*		
VCAM > 550*pg/ml*		6.56	.025	8.52	.02	1.41	–	51.58
Donor HTA		2.4	.175	2.56	.194	0.62	–	10.61
Donor BMI >30		3.5	.071	4.24	.071	0.88	–	20.37

**Table:** 

*CKD-EPI at 3 months post-transplant < 45* mL/min/1.73 m^2^ *n = 63*	*Univariate analysis*		*Multivariate linear regression*				
	*OR*	*p*	*OR*	*p*	*95% CI*		
VCAM > 550*pg/ml*	7.04	.001	4.04	.034	1.11	–	14.73
Donor HTA	4.67	.006	2.58	.151	0.71	–	9.42
Donor age > 58 years	6.75	.001	2.54	.190	0.63	–	10.19

HTA: hypertension, p values ≤.05 were considered as significant. A bivariate logistic regression allowed us to estimate crude odds ratio (left panel). Significant variables, based on a threshold p value ≤.2 in univariate analysis were introduced in a multivariate logistic regression to assess the independent associations of clinico-biological characteristics with early graft dysfunction by day 7 post-transplant surgery (primary endpoint) in the entire cohort (upper table n = 74) or in the ECD cohort (n = 47). The lower table shows the univariate and multivariate analysis of parameters associated with the secondary endpoint (eGFR at 3 months < 45mL/min/1.73 m^2.^ Adjusted odds ratios and their 95% confidence intervals were estimated.

We further aimed to evaluate whether other inflammatory factors released in the donor perfusion fluid can associate with kidney transplant dysfunction function later on at 3 months post-transplantation. Multivariate analysis identified VCAM values >550 pg/ml as the only independent factor associated with persistent CKD (DGF <45mL/min/1.73 m^2^) at 3 months post-transplant, independently of other risk factors such as donor HTA and aging (**Table 4**).

## Discussion

Early graft dysfunction remains one of the most challenging complications in kidney transplantation. Inflammation in the early phase after kidney transplantation has been reported to be associated with increased long-term all-cause mortality (20). This prospective single-center study investigated whether assessment of inflammatory biomarker levels in the kidney transplant preservation fluid during pre-transplant cold storage could be of value to predict early kidney transplant dysfunction. Our central hypothesis was that inflammatory cytokines, chemokines or leucocyte adhesion molecules could reflect donor-related injurious stimuli that render transplant vasculature more susceptible to ischemia reperfusion injury (IRI), thus affecting early dysfunction of the transplant. While as previously described, sICAM-1 levels were comparable among donors (21), a significant correlation between VCAM levels and early graft dysfunction was revealed in this study. This association would be more likely to occur during cold ischemia of long duration. Indeed, the levels of VCAM in perfusate were significantly increased over time, notably in the ECD group, indicating continuous release of this molecule from the isolated kidney during cold storage.

In addition, our results suggest that elevated VCAM levels are associated with upregulation of other endothelial inflammation markers such as fractalkine, ICAM and IL6-R. In line with our previous data that identified an inverse correlation between NK cell infiltrate and angiogenic activity (18), enhanced VCAM levels were also associated with reduced angiogenic activity and higher representation of inflammatory NK cells in the stromal vascular fraction of the transplant perirenal adipose tissue, which have been previously associated with donor aging (18). While donor age is a major factor related to kidney dysfunction, recent studies suggest that allograft survival of kidney from elderly donors (≥ 65 years) with AKI is acceptable and may be considered to enhance the donor pool for aging recipients (22, 23).This original and non-invasive approach to refine assessment of the inflammatory and vascular state of marginal transplants could have clinical implications both for advancement in the understanding of the mechanisms involved in renal ischemia-reperfusion injury and in the designs of preventive strategies. Providing development of a rapid molecular diagnostic tool, this biomarker approach could enable a more accurate assessment of the quality of donor kidney, thus potentially reducing graft dysfunction incidence.

To date, the utility of cold organ storage preservation fluid as a tool to assess the viability of kidneys for transplant remains unclear. Levels of GM-CSF and leptin in donor kidney preservation fluid have been identified as potential markers predicting short-term post-transplantation kidney function (24). Recent reviews report the potential of imaging or new technologies allowing assessment of donor-related biomarkers that could reflect graft quality (7, 9). Donor-related biomarkers have been described in HMP perfusate and in donor urine (11), and have identified glutathione S-transferase (GST) and lactate dehydrogenase (LDH) elevation as the most studied and promising biomarkers to predict short-term graft function. GST was the best biomarker for predicting DGF, but its predictive value was at best moderate. There is a need to determine whether perfusate biomarkers produced during cold storage can predict post-transplant outcomes and assess the suitability of organs for transplantation.

To our knowledge, our study is the first to report a significant correlation between VCAM levels in the perfusate and early kidney graft dysfunction. Moreover, compared with previous studies that analyzed conservation fluid biomarkers, VCAM appears to retain higher diagnostic accuracy to predict early graft dysfunction. These results are in line with observations obtained after lung transplantation that reported soluble VCAM measured in the lung perfusate was significantly associated with primary graft dysfunction (25). Several studies have suggested that induction of adhesion molecules is an underlying mechanism of acute renal injury and destruction of capillaries and tubular cells in rejection kidneys (26).

From a mechanistic point of view, our results obtained on transplant perirenal adipose tissue suggest that elevated VCAM could be associated with lesser angiogenic activity of endothelial cells in the marginal donor microenvironment and reflect enhanced adhesion and recruitment of inflammatory NK cells in aging donors that associate with subsequent kidney allograft dysfunction. Several studies have suggested that induction of adhesion molecules is an underlying mechanism of acute transplant injury. Renal IRI is a common physiopathological process in patients with kidney transplants and is associated with delayed graft function, graft rejection, and chronic graft dysfunction (27). VCAM, which is expressed by endothelial and antigen-presenting cells, appears to play a key role in renal IRI. Gibbs et al. were the first to report that induction of VCAM during kidney graft dysfunction could contribute to the recruitment of mononuclear cells and make renal endothelial and tubular cells more susceptible to cell-mediated injury.^20^ The interactions of VCAM-1 with its VLA-4 receptor, expressed by lymphocytes, macrophages and granulocytes, initiate immune inflammation and responses to alloantigen in the early development of acute and chronic rejection (28, 29). VCAM is therefore considered as a marker of endothelial activation and vascular inflammation. Some studies have also revealed an impact of ICAM1 and VCAM1 gene polymorphisms on delayed graft renal function (30). A significant correlation between higher VCAM expression on human kidney biopsy specimens obtained during transplant surgery and delayed graft function and high serum creatinine levels at 1 and 3 years after transplantation in the recipients has been previously reported (31).

Our study also further supports that risk factors associated with marginal donors, aging, hypertension, and a history of stroke could further sustain fractalkine-mediated endothelial activation, leucocyte recruitment and impact transplant vascular injury and repair (32). The fractalkine-CX3CR1 pathway has also been identified in the molecular signature of transplant dysfunction in biopsies (10, 33). A trend associating soluble IL6 receptor levels and IL6R/IL6 ratios was also observed in donor conservation fluid from ECD patients and could refine personalized assessment of vascular risk in these marginal donors. IL6-driven endothelial inflammation is a well-documented axis associated with cardiovascular risk after kidney transplantation and blockade of the IL-6/IL6-R signaling pathway has been evoked as a means to limit the harmful effects of inflammation in experimental studies Our study also further supports that risk factors associated with marginal donors such as aging, hypertension, and a history of stroke could further sustain fractalkine-mediated endothelial activation and leucocyte recruitment and impact transplant vascular injury and repair and kidney and heart transplant rejection (34, 35). Our observations suggest that anti-inflammatory treatment targeting the VCAM and fractalkine pathways could antagonize recruitment of activated leucocytes during pre-transplant perfusion or in the recipient in order to improve renal function recovery.

These results could also be relevant to improve vascular resistance of ECD transplants. Intensive research has focused on the development of innovative and dynamic preservation techniques in order to limit IR vascular damage and promote regeneration through pharmacological intervention. The administration of pharmacological agents during cold storage or during the post-transplant period in recipients offers a window of opportunity to treat and optimize the quality of the graft. For instance, a pilot randomized clinical trial showed administration of 600mg N-acetylcysteine, twice a day versus placebo, to kidney transplant recipients resulted in better renal function throughout the first 90 days and at 1 year and the risk of DGF was significantly lower (36). Anti-VCAM-1 and anti-VLA-4 antibodies synergistically block rejection of transplanted organs (37, 38).

The present study has some limitations that should be acknowledged. Due to the potential associated morbidity (24), no biopsy of the graft was performed in our study and we are consequently unable to make a strict correlation between preservation solution VCAM level and VCAM tissue gene expression. This study is also limited by its small sample size that warrants further validation of these biomarkers in larger cohorts. Nevertheless, multiple logistic regression analyses support the view that VCAM can be identified as a valuable soluble adhesion molecule reflecting donor-related inflammatory features that associate with dysfunction of the transplant. In contrast with zero-hour biopsies, the noninvasive evaluation of these soluble biomarkers could be achieved in donor kidney preservation fluid before transplantation using ELISA- or Luminex-based assays and provide easy-to-implement methods predicting vascular transplant quality and early kidney function. As previous studies have suggested, removal-dilution of proinflammatory cytokines during HMP could reduce production of chemokines and adhesion molecules such as ICAM-1 (27).

While larger scale and pre-clinical studies will be needed, our study could open perspectives to antagonize VCAM or Fractalkine/IL-6 driven activation pathways during organ perfusion in order to limit endothelial dysfunction prior to transplant.

In conclusion, in this prospective pilot study, we found that VCAM levels in the cold organ storage preservation fluid could recapitulate the inflammatory risk associated with age and HTA and be identified as a non-invasive independent predictor of early kidney graft dysfunction after kidney transplantation. These results strengthen the implication of VCAM in renal IRI. Targeting this biological pathway could be one of the promising therapeutic strategies to improve the quality of grafts from ECD donors.

## Data availability statement

The original contributions presented in the study are included in the article/**Supplementary Material**. Further inquiries can be directed to the corresponding author.

## Ethics statement

The study protocol was approved by the National Ethics Committee of the Agence de la Biomédecine (PFS18-013), the National Ministry of Research and adhered to the Jardé Law on human investigation. The patients/participants provided their written informed consent to participate in this study.

## Author contributions

Study concept and design: PP, RB, and AD. Acquisition of data: MB, BG-T, PA, LL and PF. Analysis and interpretation of data: MB, BG-T, PP, and AD. Drafting of the manuscript: MB, AD, and PP. Critical revision of the manuscript for important intellectual content: EL, RB, and FS. Statistical analysis: BG-T and PP. All authors contributed to the article and approved the submitted version.

## Funding

The study was funded by the French Urological Association “*Association Française d’Urologie”* and the EAU Section of Transplantation Urology (ESTU). The funders of the study were not involved in overall study management.

## Conflict of interest

All authors declare that the research was conducted in the absence of any commercial or financial relationships that could be construed as a potential conflict of interest.

## Publisher’s note

All claims expressed in this article are solely those of the authors and do not necessarily represent those of their affiliated organizations, or those of the publisher, the editors and the reviewers. Any product that may be evaluated in this article, or claim that may be made by its manufacturer, is not guaranteed or endorsed by the publisher.

## Abbreviations

ECD, Extended criteria donors; SCD, Standard criteria donor; DGF, Delayed graft function; SGF, Slow graft function; HMP, Hypothermic machine perfusion; KPS-1, Kidney preservation solution 1; IL-6, Interleukin 6; IL-6R, IL-6 receptor; TNFα, Tumor Necrosis Factor; ICAM, InterCellular Adhesion Molecule; VCAM, Vascular Cell Adhesion Molecule; CXCL1, C-X-C Motif Chemokine Ligand 1; IFNγ, Interferon-γ; CX3CL1 or Fractalkine, C-X3-C motif ligand 1; CKD-EPI, Chronic Kidney Disease Epidemiology Collaboration; VIF, Variance Inflation Factors; eGFR, Estimated Glomerular Filtration Rate; IRI, Renal ischemia-reperfusion injury; GST, Glutathione S-transferase; LDH, Lactate dehydrogenase.
