# Supplementary material for: An enhanced level of VCAM in transplant preservation fluid is an independent predictor of early kidney allograft dysfunction
Source: Front Immunol. 2022 Aug 11;13:966951. doi: 10.3389/fimmu.2022.966951 (PMC9403542; doi:10.3389/fimmu.2022.966951)
Supplement: Supplementary file 1 [file Table_1.docx]

**Supplemental Table1: Analysis of the correlation between creatinine at Day7 post-transplant and the evolution of renal function during the first year of transplantation**

| **Creatinine at Day 7 post-Transplant** | **n** | **Spearman r** | **p value** |
| --- | --- | --- | --- |
| Creatinine Day 6 | 74 | 0.953 | <.0001 |
| Creatinine Day 5 | 74 | 0.946 | <.0001 |
| Creatinine Day 4 | 74 | 0.91 | <.0001 |
| Creatinine Day 3 | 74 | 0.893 | <.0001 |
| Creatinine Day 15 | 72 | 0.884 | <.0001 |
| Creatinine Day 21 | 72 | 0.755 | <.0001 |
| Creatinine Day 30 | 72 | 0.713 | <.0001 |
| Creatinine Day180 | 44 | 0.71 | <.0001 |
| Creatinine Day 210 | 43 | 0.709 | <.0001 |
| Creatinine Day 45 | 69 | 0.695 | <.0001 |
| Creatinine Day 150 | 52 | 0.685 | <.0001 |
| Creatinine Day120 | 56 | 0.66 | <.0001 |
| Creatinine Day 60 | 64 | 0.639 | <.0001 |
| Creatinine Day 360 | 41 | 0.63 | <.0001 |
| Creatinine Day 240 | 41 | 0.629 | <.0001 |
| Creatinine Day 90 | 63 | 0.583 | <.0001 |
| CKD-EPI Day 90 | 63 | -0.569 | <.0001 |
| CKD-EPI Day 60 | 64 | -0.597 | <.0001 |
| CKD-EPI Day 120 | 56 | -0.605 | <.0001 |
| CKD-EPI Day 360 | 41 | -0.609 | <.0001 |
| CKD-EPI Day 240 | 41 | -0.644 | <.0001 |
| CKD-EPI Day 150 | 52 | -0.652 | <.0001 |
| CKD-EPI Day 45 | 69 | -0.662 | <.0001 |
| CKD-EPI Day 30 | 72 | -0.675 | <.0001 |
| CKD-EPI Day 210 | 43 | -0.686 | <.0001 |
| CKD-EPI Day 180 | 44 | -0.688 | <.0001 |
| Donor Age | 74 | 0.219 | .0614 |

Serum creatinine is reported as micromoles of creatinine to a liter of blood (micromoles/L).

The Chronic Kidney Disease Epidemiology Collaboration (CKD-EPI) equation was used to estimate Glomerular filtration rate (mL/min per 1.73 m2) after the first month post transplant surgery (Day 30).

**Supplemental Table 2: Inflammatory biomarker levels evaluated by ELISA in preservation fluid from SCD and ECD transplants**

|  | Overall Cohort  (n = 74) | SCD  (n = 27) | ECD  (n = 47) | p value |
| --- | --- | --- | --- | --- |
| IL-6, pg/mL | 54.2 (19.8-118) | 55.8 (35.7-135) | 54.2 (16.8-117) | .43 |
| IL-6R, pg/mL | 519 (270-942) | 420 (228-661) | 541 (370-1044) | .06 |
| Ratio IL-6R/IL-6 | 11.9 (3.2-25) | 6 (2.7-19.2) | 12.7 (4-38) | .06 |
| TNFα, pg/mL | 4 (4-21) | 4 (4-32.6) | 4 (4-9.9) | .18 |
| ICAM, pg/mL | 1940 (510-9323) | 1620 (530-6330) | 1980 (450-9910) | .45 |
| VCAM pg/mL | 550 (500-11488) | 500 (500-500) | 8830 (500-14970) | <.0001 |
| CXCL1, pg/mL | 15.1 (8-38) | 24.9 (8.4-48.3) | 13.5 (8-32.5) | .13 |
| IFNγ, pg/mL | 1 (1-1.75) | 1 (1-2.1) | 1 (1-1) | .08 |
| Fractalkine, pg/mL | 193 (17-627) | 27.1 (10-146) | 383 (127-819) | <.0001 |

SCD: standard criteria donors; ECD: extended criteria donors. Indicated values correspond to median values and 25-75 interquartile ranges. Median values observed in SCD and ECD donors were considered significantly different in Mann-Whitney Test when p values were < .05.
